# Molecular Identification of *Sarcocystis* sp. (Apicomplexa, Sarcocystidae) in Offspring of Tengmalm's Owls, *Aegolius funereus* (Aves, Strigidae)

**DOI:** 10.3389/fvets.2021.804096

**Published:** 2021-12-24

**Authors:** Ondřej Máca, Marek Kouba, Erkki Korpimäki, David González-Solís

**Affiliations:** ^1^Department of Pathology and Parasitology, State Veterinary Institute Prague, Prague, Czechia; ^2^Department of Zoology and Fisheries, Faculty of Agrobiology, Food and Natural Resources, Czech University of Life Sciences Prague, Prague, Czechia; ^3^Department of Ethology and Companion Animal Science, Faculty of Agrobiology, Food and Natural Resources, Czech University of Life Sciences Prague, Prague, Czechia; ^4^Section of Ecology, Department of Biology, University of Turku, Turku, Finland; ^5^El Colegio de la Frontera Sur, Chetumal, Mexico

**Keywords:** birds, Europe, intestinal mucosa, molecular characterization, oocysts and sporocysts, phylogeny

## Abstract

**Background:** Birds act as intermediate or definitive hosts of cyst-forming coccidia parasites of the genus *Sarcocystis* Lankester, 1882. However, the spectrum of species of *Sarcocystis* in birds and the role of the latter in the transmission of coccidia are still incomplete for many avian species, including the Tengmalm's owl *Aegolius funereus* (Linnaeus, 1758). During the research on Tengmalm's owls in Finland, some fledglings were found dead and subsequently parasitologically examined. Therefore, this study is focused on the morphological and molecular description of a *Sarcocystis* species found in the intestine of the Tengmalm's owl and its possible role as a definitive host.

**Methods:** Eleven fledgling owls in the Kauhava region of west-central Finland were found dead and subsequently were submitted for necropsy and parasitologically examined through the flotation–centrifugation coprological technique for the presence of oocysts/sporocysts of the genus *Sarcocystis* by light microscopy. Wet mounts were used for the examination of muscle samples (breast, legs, and heart). Polymerase chain reaction (PCR) and nested-PCR were carried out using primers for 18S rRNA, 28S rRNA, ITS1 region, and CO1 genes.

**Results:** All 11 examined owls were parasitized by numerous sporocysts and oocysts in the intestinal mucosa scrapings (prevalence, 100%). Sporulated oocysts and sporocysts measured 16.34–16.96 × 11.47–12.09 μm and 11.85–13.52 × 7.77–9.25 μm, respectively. The skeletal and heart muscles were negative for sarcocysts. *Sarcocystis* sp. ex *Aegolius funereus* (hereafter *Sarcocystis* sp. Af) is closely related to *Sarcocystis strixi* in the barred owl (*Strix varia* Barton, 1799) from the USA and *Sarcocystis* sp. isolate 5 in the European shrew (*Sorex araneus* Linnaeus, 1758) from the Czech Republic. Phylogenetic analysis allowed determining the relationship of the herein reported *Sarcocystis* sp. with its congeners.

**Conclusions:** This work provided the first and most comprehensive record on *Sarcocystis* from owls obtained in Finland, thus highlighting the importance of molecular data in species identification.

## Introduction

Cyst-forming coccidia parasites of the genus *Sarcocystis* Lankester, 1882, can infect a wide variety of vertebrates, including birds, which could act as definitive and intermediate hosts in the life cycle of these parasites. However, the spectrum of species of *Sarcocystis* in birds and the role of the latter in the transmission of coccidia are still incomplete for many avian species, including the Tengmalm's owl *Aegolius funereus* (Linnaeus, 1758). This species is a small nocturnal cavity-nesting owl living in coniferous forests in the boreal zone and alpine forests further south in the Holarctic region (1, 2). It feeds mainly on small mammals, among which voles of the genera *Myodes* Pallas, 1779 (=*Clethrionomys* Tilesius, 1850) and *Microtus* Schrank, 1798, are their main prey, while shrews of the genus *Sorex* and small forest birds are their most important alternative prey items (3–5).

To date, relatively few studies have been conducted on the *Sarcocystis* species in *A*. *funereus* in wild; in fact, only Wiesner (6), in a scientific meeting, reported sporocysts of *Sarcocystis* sp. in the Tengmalm's owl, which were experimentally developed in the bank vole *Myodes glareolus* (=*Clethrionomys glareolus*) Schreber, 1780. Whereas Zuo et al. (7) and Zuo and Yang (8) were unsuccessful in experimentally infecting *A*. *funereus* with *Sarcocystis sinensis* Zuo, Zhang et Yie, 1990, from China.

During radio telemetry research of Tengmalm's owls in Finland, where decreasing densities of the main prey (voles) occurred, some fledglings were found dead and subsequently parasitologically examined. Since this owl species has practically no records of species of *Sarcocystis* and the role of owls in the life cycle of the parasite is partially known, this study is focused on the morphological and molecular description of a *Sarcocystis* species found in the intestine of the Tengmalm's owl and its possible role as definitive host.

## Materials and Methods

The carcasses of 11 specimens (29–47 days old from hatching, 98–136 g in body weight) from 7 different nests (10 died due to starvation and infection and 1 due to pine marten, *Martes martes* Linnaeus, 1758, predation) (MK and EK, unpublished data) were examined in this study. They were collected in the Kauhava region of west-central Finland (63°N, 23°E) during a radio-tracking study of Tengmalm's owl fledglings during the post-fledging dependence period in 2019. The study area is located 30–120 m above sea level and mostly covered by forest [for a detailed description of the study area, see (4, 9, 10)]. The aerial distances (*n* = 21) between involved nest boxes from which the fledglings originated and later subjected to necropsy were 19.2 ± 9.3 km on average (range = 3.8–38.7 km).

Necropsies were carried out at the State Veterinary Institute (SVI) Prague, Czech Republic, where the intestinal content and muscular samples (breast, legs, and heart) of thawed birds were parasitologically examined in wet mounts using water or glycerin for orientation purposes. After parasite detection, intestinal mucosa scrapings were used for the flotation–centrifugation coprological method under light microscopy for the final evaluation and presence of oocysts/sporocysts using a Leica DMLB optical microscope with a Leica DFC420 digital camera (Leica Microsystems, Wetzlar, Germany) and isolation to Eppendorf tubes for DNA extraction. All measurements are given in micrometers, unless otherwise mentioned.

Genomic DNA was extracted by glass bead disruption from 22 isolates (two from each owl) of oocysts/sporocysts using the QIAamp® Fast DNA Stool Mini Kit (Qiagen, Hilden, Germany) according to the manufacturer's recommendations. DNA was stored at −20°C until further use.

Polymerase chain reaction (PCR) and nested-PCR were carried out using primers for 18S rRNA (ERIB1/A2R, A1F/S2r, A2F/Primer BSarc and Fext/Rext, and Fint/Rint, respectively) (11–15), 28S rRNA (KL_P1R/KL_P1F) (16), internal transcribed spacer region 1 (ITS1 region) (ITS-F/ITS-R) (16), and cytochrome c oxidase subunit 1 (CO1) genes (SF1/SR10) (17, 18), with recommended PCR annealing temperature based on the primer pairs used. Each PCR mixture contained 12.50 μl of GoTaq® G2 Green Master Mix (Promega, Madison, WI, USA), 0.4 μM of each primer, 5 μl DNA template, and nuclease-free water to a total volume of 25 μl. The PCR conditions consisted of initial denaturation at 95°C for 5 min, followed by 35 cycles of 95°C for 30 s, 52–60°C for 30 s, 72°C for 1 min, and then a final extension step at 72°C for 10 min. The amplification products were resolved on 1.5% agarose gels and visualized by ethidium bromide staining. The PCR products were purified using the ExoSAP-IT™ Express PCR Product Cleanup Reagent kit (Thermo Fisher Scientific, Waltham, MA, USA) according to the manufacturer's protocol. Purified PCR products were directly sequenced in both forward and reverse directions using the same primers as for PCR through the commercial company Eurofins Genomics (Ebersberg, Germany). Representative nucleotide sequences of 18S rRNA, 28S rRNA, ITS1, and the CO1 loci of *Sarcocystis* sp. Af have been deposited in GenBank under the accession numbers MW349706, MW349707, MW373964, and MW489293, respectively. Sequences from both forward and reverse strands were assembled and manually edited using FinchTV software (Geospiza Inc., Seattle, WA, USA), followed by BLAST (Basic Local Alignment Search Tool) program at the NCBI (National Center for Biotechnology Information) server. Searches were conducted on the obtained sequences for genus/species identification. The sequence chromatograms obtained in this study were aligned using MAFFT software, version 7 (19).

Phylogenetic trees for all datasets were generated from the nucleotide sequences of the selected *Sarcocystis* species using MEGA X (20) and reconstructed using the neighbor-joining (NJ) and maximum likelihood (ML) methods. A NJ phylogenetic tree for the 18S rRNA gene (dataset with 25 nucleotide sequences with a total of 1,644 aligned nucleotide positions) was computed according to the Tamura–Nei model with a gamma distribution (TN93+G). Other phylogenetic trees were generated using ML analyses based on the Kimura two-parameter model with a gamma distribution rate and a proportion of invariant sites (K2+G+I) for the 28S rRNA gene (25 sequences with 1,442 positions). For the CO1 gene (18 sequences with 1,013 positions), the Hasegawa–Kishino–Yano model with a gamma distribution (HKY+G) was used, while the Tamura–Nei model with a gamma distribution rate and a proportion of invariant sites (TN93+G+I) was used for the ITS1 region (24 sequences with 1,426 positions). All four phylogenetic trees were rooted using the *Toxoplasma gondii* sequence. Consensus trees were obtained after bootstrap analysis with 1,000 replications.

## Results

All intestinal samples of the 11 owls examined under a light microscope were positive to oocysts and sporocysts of *Sarcocystis*, whereas samples of muscular tissues were negative. The oocysts/sporocysts were described as follows:

Family Sarcocystidae Poche, 1913

*Sarcocystis* sp. ex *Aegolius funereus*

**Figure 1:**
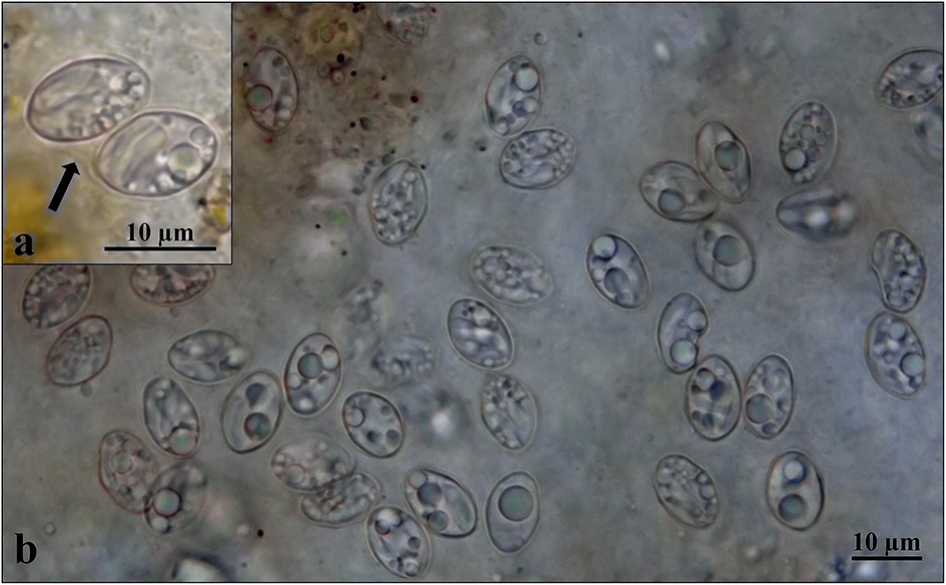
Light micrographs of oocyst **(a)** and numerous sporocysts **(b)** in the intestinal mucosa. *Arrow* indicates thin oocyst wall.

Description: thin-walled sporulated oocysts, 16.34–16.96 × 11.47–12.09 (*n* = 5), and sporocysts were 11.85–13.52 × 7.77–9.25 (*n* = 35) (Figures 1a,b).

*Taxonomic summary*

Definitive host: Tengmalm's owl *Aegolius funereus* Linnaeus, 1758 (Strigiformes: Strigidae)

Intermediate host: Unknown

Distribution: Kauhava region, west-central Finland (~63°N, 23°E)

Site of infection: Small intestine

Prevalence: 100% (11 owls examined/11 infected)

Deposited material: Symbiotype (oocysts/sporocysts in 2.5% potassium dichromate); genomic DNA in Eppendorf tubes stored at SVI Prague.

GenBank sequences: MW349706 (18S rRNA gene), MW349707 (28S rRNA gene), MW373964 (ITS1 region), and MW489293 (CO1 gene).

All primers worked properly and generated genetic sequences of 20 *Sarcocystis* isolates from 10 birds (isolates from one bird failed), which were obtained for the 18S rRNA, 28S rRNA, ITS1, and CO1 loci, of which those of six owls were identified as *Sarcocystis* sp. Af, while those from four and one owls were excluded due to their low quality/short fragments of sequences or failure in amplifying, respectively. All obtained 18S rRNA sequences were identical; therefore, only one of 1,773 bp was submitted to GenBank (MW349706). It shared 99.69% similarity with an *Sarcocystis* sp. isolate 5 (as named in GenBank) (1,594 bp; AF513487) in the European shrew (*Sorex araneus* Linnaeus, 1758) from the Czech Republic; 99.61% with *S*. *strixi* Verma, von Dohlen, Mowery, Scott, Cerqueira-Cézar, Rosenthal, Dubey et Lindsay, 2017 (MF162315), in the barred owl (*Strix varia* Barton, 1799) from the USA; 99.55% with *Sarcocystis corvusi* Prakas, Kutkiené, Butkauskas, Sruoga et Žalakevičius, 2013 (JN256117), in the jackdaw [*Corvus monedula* (Linnaeus, 1758)] from Lithuania, *Sarcocystis halieti* Gjerde, Vikøren et Hamnes, 2018 (MH130211 and MF946587), in the great cormorant [*Phalacrocorax carbo* (Linnaeus, 1758)] from Lithuania, and the white-tailed sea eagle [*Haliaeetus albicilla* (Linnaeus, 1758)] from Norway; and 98.82% with *Sarcocystis dispersa* Cerná et Sénaud, 1977, in the barn owl *Tyto alba* (Scopoli, 1769) from the Czech Republic. The representative 28S rRNA sequence (MW349707) was 1,509 bp and shared 97.59% genetic similarity with *S*. *strixi* (MF162316) and *Sarcocystis lari* Prakas, Kutkiené, Butkaukas, Sruoga et Žalakevičius, 2014 (MF946611), in the white-tailed sea eagle from Norway, whereas it shared 97.49% similarity with *S*. *lutrae* Gjerde et Josefsen, 2015 (KM657771), in the Eurasian otter [*Lutra lutra* (Linnaeus, 1758)] from Norway and 96.22% with *Sarcocystis* sp. isolate 5 (555 bp; AF513497), although it was not included in the phylogenetic tree due to its short sequence. Analyses of the CO1 gene sequences (MW489293, 1,060 bp) showed a high similarity (99.52%) with *S*. *strixi* (MF162317), 99.43% with *S*. *lutrae* (KM657808), and 99.42% with *S*. *lari* (MF596283 and MF946584) in the great black-backed gull [*Larus marinus* (Linnaeus, 1758)] from Lithuania and the white-tailed sea eagle from Norway. Representative nucleotide ITS1 region sequence was 1,294 bp (MW373964), with no significant match to other *Sarcocystis* species from GenBank, except 89.57% similar (44% query cover) to *S*. *halieti* (MF946596) and 89.52% similar (35% query cover) to *S*. *lutrae* (MG372109) in *L*. *lutra* from the Czech Republic. Single cases of double peaks were noted at 28S rRNA at nucleotide positions 666 and 667 (TT/CC), while some ITS1 sequences differed only by one single nucleotide polymorphism (T/C) at nucleotide position 466.

**Figure 2:**
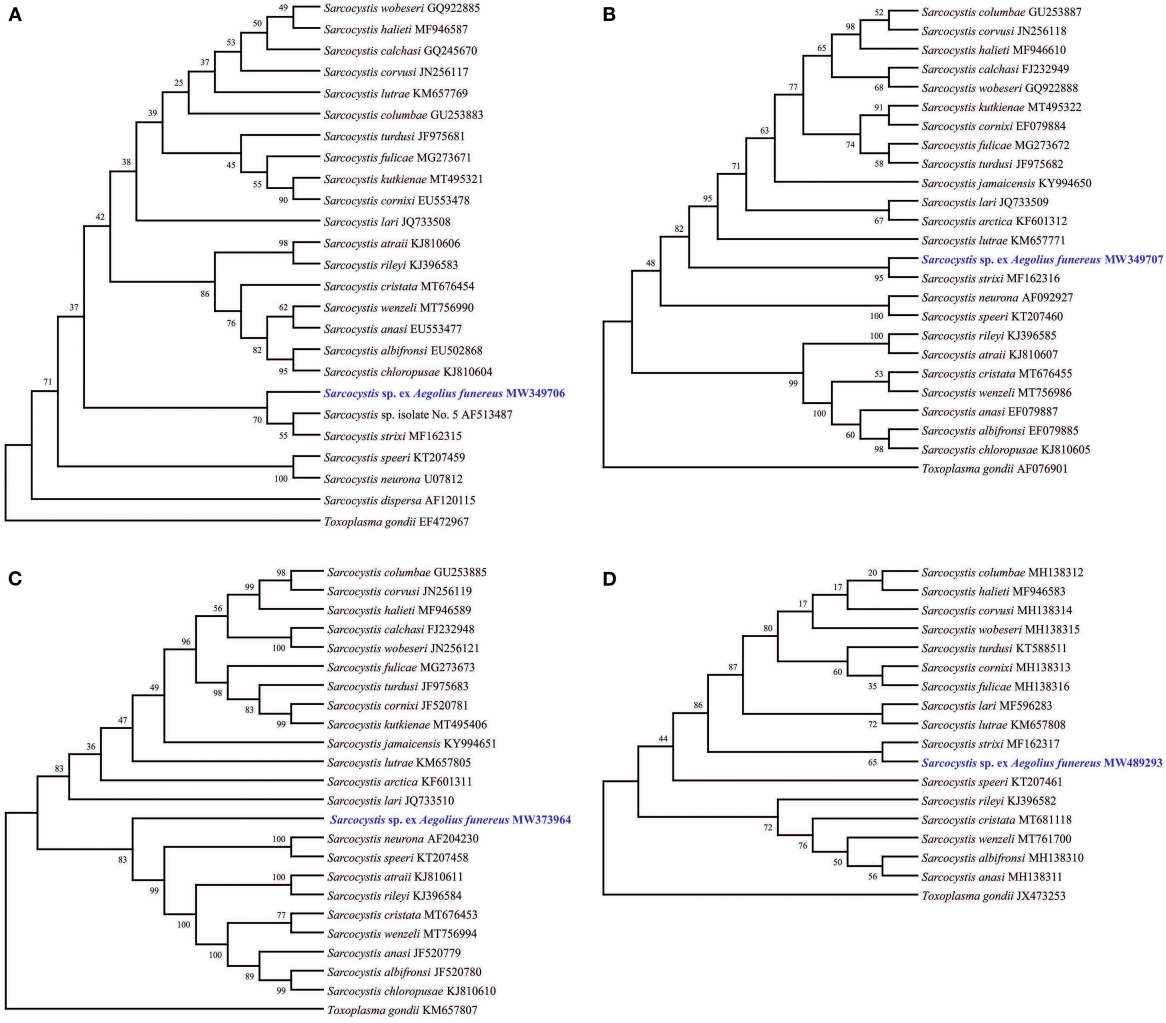
Phylogenetic trees of the species of *Sarcocystis* from various hosts based on sequences of 18S rRNA **(A)**, 28S rRNA **(B)**, ITS1 **(C)**, and CO1 **(D)** loci. The *numbers on phylogenetic trees* represent bootstrap values based on 1,000 replications. Genbank accession numbers follow the *Sarcocystis* taxa.

The phylogenetic trees showed different topologies and relationships between *Sarcocystis* sp. Af with its congeners according to the availability of sequences. Phylogenetic trees based on 18S rRNA, 28S rRNA, and CO1 genes showed a clade formed by parasites using owls as definitive hosts and rodents as intermediate hosts, such as *Sarcocystis* sp. Af, *S*. *strixi*, and *Sarcocystis* sp. 5 (in the case of 18S rRNA) (Figures 2A,B,D), while the tree of the ITS1 region showed *Sarcocystis* sp. Af in a single clade since the ITS1 sequence of *S*. *strixi* was not available, although the former formed a group with other *Sarcocystis* spp. derived from birds and terrestrial carnivores (Figure 2C).

## Discussion

The first published finding and description of the oocysts/sporocysts of *Sarcocystis* sp. in the Tengmalm's owl was made by Wiesner (6) in Europe. Other *Sarcocystis* using owls as definitive hosts are *Sarcocystis espinosai* (Espinosa, Sterner, Blixt et Cawthorn, 1988) Odening, 1997, in the northern saw-whet owl *Aegolius acadicus* (Gmelin, 1788) from the USA (21); *S*. *dispersa* in the long-eared owl *Asio otus* (Linnaeus, 1758), barn owl *T*. *alba*, masked owl *Tyto novaehollandiae* (Stephens, 1826), and *Ninox novaeseelandie* (Gmelin, 1788) from the Czech Republic and Australia (22–26); *Sarcocystis rauschorum* Cawthorn, Gajadhar et Brooks, 1984, in the snowy owl *Bubo scandiacus* (reported as *Nyctea scandiaca*) (Linnaeus, 1758) from Canada (26); *Sarcocystis scotti* Levine et Tadros, 1980 [this species was considered invalid; see (27)], and *Sarcocystis sebeki* (Tadros et Laarman, 1976) Levine, 1978, both in the tawny owl *Strix aluco* Linnaeus, 1758, from Europe (28, 29) and *S*. *strixi* in the barred owl *Strix varia* from the USA (30). Hoberg et al. (31) reported a coccidian (resembling *Frenkelia* or *Sarcocystis*) in the northern spotted owl *Strix occidentalis caurina* Xantus de Vesey, 1860, from the USA, although the proper identity of the parasite was undetermined. There also are reports of owls acting as intermediate hosts for *Sarcocystis falcatula* Stiles, 1983, in the great-horned owl *Bubo virginianus* (Gmelin, 1788) from the USA (32) and *Sarcocystis otus* Krone, Rudolphi et Jakob, 2000 [invalid species according to Dubey et al. (27)] in *A. otus* from Germany (33). Most of these were solely morphologically studied, while only sporocysts of *S*. *dispersa* in *T*. *alba* (18S rRNA) (34) and *S*. *strixi* (18S rRNA, 28S rRNA, and CO1) (30) were morphologically and molecularly characterized.

The sizes of fully sporulated sporocysts of *S*. *espinosai, S*. *rauschorum, S*. *sebeki*, and *S*. *strixi* are within the range for those of *Sarcocystis* sp. Af (9.5–14.0 × 8.0–12.0, 9.6–14.0 × 7.0–10.0, and 11.2–13.7 × 8.8–10.9 μm vs. 11.8–13.5 × 7.7–9.2 μm) and partially with the sporocysts and oocysts of *Sarcocystis* sp. in *S*. *o*. *caurina, S*. *dispersa*, and *S*. *sebeki* (11.0–12.0 × 5.0–6.0, 11–14 × 8–11, and 10.0 × 14.0 μm vs. 11.8–13.5 × 7.7–9.2 μm; 12.4–15.5 × 9.3–12.4 and 17–20 × 10–13 μm vs. 16.3–16.9 × 11.4–12.1 μm). Since the morphological parameters of oocysts/sporocysts are insufficient to distinguish species, the comparison of these *Sarcocystis* with *Sarcocystis* sp. Af is unreliable. The sporocysts and oocysts of *Sarcocystis* sp. of Wiesner (6) were not described, so it is impossible to say whether it belongs to *Sarcocystis* sp. Af, although they could be conspecific.

On the other hand, of those species molecularly characterized, such as *S*. *dispersa* (18S rRNA), *S*. *strixi* (18S rRNA, 28S rRNA, and CO1), and *Sarcocystis* sp. 5 (18S rRNA), the first formed a different clade from that of *Sarcocystis* sp. Af, while the second grouped with *Sarcocystis* sp. Af in 28S rRNA and CO1 genes, and formed a branch with the third. Apparently, *S*. *strixi* and *Sarcocystis* sp. 5 are closely related (sister) to *Sarcocystis* sp. Af, but with genetic differences to be still considered as separated species, although this confirmation should wait until the ITS1 sequences of these two taxa are known and compared using the most genetic marker. Unfortunately, the ITS1 region was not used previously in *S*. *dispersa* or in *S*. *strixi*, thus making their comparison with *Sarcocystis* sp. Af limited. The use of various loci has been widely applied in several papers [e.g., (15, 30, 35)] to compare known and new species that were partially or completely genetically analyzed. This fact has helped in determining that the ITS1 region is more sensitive to the genetic differences among *Sarcocystis* species from birds and carnivores as intermediate hosts [see (36–38)], while the 18S rRNA, 28S rRNA, and especially CO1 (commonly <1%) genes are now considered of limited taxonomic help (15, 27, 35). For example, *S. lari* (JQ733508, JQ733509, MF596283, and JQ733510) and *Sarcocystis wobeseri* (GQ922885, GQ922887, MH138315, and GU475111) are very similar to each other at 18S rRNA (99.67%), 28S rRNA (98.74%), and CO1 (99.62) genes, but differ at ITS1 (75.89%); thus, both are considered as different species. In the present case, *Sarcocystis* sp. Af and *S. strixi* are more different at 18S rRNA (99.61%), 28S rRNA (97.59%), and CO1 (99.52%) than the above-mentioned species, but since the ITS1 sequence of *S*. *strixi* is missing, the specific differentiation of both species is possible. In fact, *S. corvusi* and *S. halieti* were very distant from the clade formed by *Sarcocystis* sp. Af, *S. strixi*, and *Sarcocystis* sp. 5 in the phylogenetic tree based on 18s rRNA despite the great similitude of *Sarcocystis* sp. Af with *S. corvusi* and *S. halieti* (>99%), thus confirming the limitation of this gene. Indeed, the ITS1 region varies considerably more than the sequences of the 18S and 28S rRNA genes in *Sarcocystis* spp., which parasitize birds as intermediate hosts (18, 35, 39, 40). In addition, several *Sarcocystis* species that use birds or mammalian carnivores as intermediate hosts differ very little and seem to possess little or no intraspecific variation at the CO1 marker, unlike those using ruminants as intermediate hosts; therefore, the ITS1 marker seems better suited than CO1 to separate closely related *Sarcocystis* species that use birds and carnivores as intermediate hosts [see (15, 41)].

The intermediate host of *Sarcocystis* sp. Af is unknown, but apparently rodents (different species of voles and mice inhabiting the study area) [see (4, 42)] play that important role. Experimentally, Wiesner (6) observed that the bank vole *M. glareolus* acts as an intermediate host, and it could also be the potential host for *Sarcocystis* sp. Af, while the northern saw-whet owl *A*. *acadicus*, a congeneric owl species from the USA, used the deer mice (*Peromyscus maniculatus*) [see (21)]. According to Mikkola (1) and König and Weick (2), there are more than 47 mammalian and 66 bird species used as prey by owls in Europe, which could act as intermediate hosts for *Sarcocystis* sp. Af. The most common small mammals used as prey by the Tengmalm's owl are the bank vole, field vole [*Microtus agrestis* (Linnaeus, 1761)], sibling vole [*Microtus levis* (syn. *M*. *rossiaemeridionalis*) Miller, 1908], and harvest mouse [*Micromys minutus* (Pallas, 1771)]; less commonly are shrews (genus *Sorex*) (4, 42). In the study area, the main prey of fledglings and adult Tengmalm's owls are bank voles, field voles, and sibling voles, whose abundances regularly fluctuate in high-amplitude (100- to 200-fold) 3-year cycles (43–46). Accordingly, the abundances of individual vole species fluctuate strongly. The overall prey abundance could be 0.2–13.1 and 0.6–28.2 vole individuals per 100 trap nights, as revealed by regular long-term snap trapping in the study area during spring and autumn, respectively, thus differing up to 65-fold between different years/phases of the vole cycle (4, 10, 45).

It has been mentioned that species of *Sarcocystis* forming sarcocysts in birds are less intermediate host specific than are those using mammalian intermediate hosts (15), whereas the latter are less definitive host specific, especially those using rodents as intermediate hosts [see (27)]. In the case of *M. glareolus*, it has been found as an intermediate host of several types and unnamed species of *Sarcocystis* from the Czech Republic (47), Baltic region (48), and Lithuania (49), as well as of *Sarcocystis clethrionomyelaphis* Matuschka, 1986, in Germany, which uses canids, mustelids, snakes, or birds of prey as definitive hosts [see (50, 51)]. One of the forms from Lithuania showed similar features (dense hair-like projections on the cyst wall) to that of the *Sarcocystis* sp. described by Wiesner (6) [see (49)], thus corroborating the role of the bank vole in the life cycle of the parasite. In the present case, even though the intermediate host is unknown, the molecular analysis indeed determined that the developmental stages in the definitive host belong to *Sarcocystis* sp. Af, as herein stated.

The cause of death of Tengmalm's owls was undetermined, but the occurrence of *Sarcocystis* in these birds should be monitored since other taxa of this genus (*S*. *falcatula* and *Sarcocystis* sp. isolate from chicken) have been reported as causing encephalitis in free-ranging great-horned owls (*B*. *virginianus*) and meningoencephalitis in chickens, respectively [see (32, 52)].

In the last decades, integrative taxonomy using morphological features and molecular analysis has uncovered the huge diversity of species in various groups of organisms, including protists (27). Additionally, it particularly improved the recognition of the specificity of *Sarcocystis* in their intermediate and definitive hosts around the world. Apparently, *A*. *funereus* acted as a natural definitive host of this parasite, thus representing the first host record in *A*. *funereus* and the ninth owl species with a *Sarcocystis* species. Interestingly, the Tengmalm's owl was experimentally infected with *S*. *sinensis*, but sporocysts and oocysts were not found after some days of infection [see (8)]; this could indicate the host specificity of *Sarcocystis* sp. Af. However, birds of prey might be infected by more than one *Sarcocystis* species, such as *S*. *halieti* and *S*. *lari* in the white-tailed sea eagle (*H*. *albicilla*) [see (15)]; therefore, more Tengmalm's owls, other owl species, and birds of prey should be examined to determine the presence of other species or forms.

Tengmalm's owls are nomadic, and the natal dispersal movements of juvenile owls hatched in Finland could extend more than 1,000 km (53, 54), while adult females show long-distance breeding dispersal up to >600 km in Finland (4, 53) and adult males are usually resident after their first breeding attempt (4). They can also move over long distances and are widely distributed in North and Central Europe, including the Italian Alps and the Pyrenees in North Spain (1). Therefore, it is highly probable that Tengmalm's owls could spread *Sarcocystis* sp. Af out of Finland to various other locations within their distribution range. For instance, during a long-term study of Tengmalm's owl in the Czech Republic (years 2010–2012 and 2015), a prevalence of 40% was found for a *Sarcocystis* sp. in 10 fledglings (55, 56). Thus, these parasites seem to be present in that country, although the species identification should be confirmed to determine the real distribution of *Sarcocystis* sp. Af. However, Svobodová (57) examined two Tengmalm's owls in the Czech Republic, which were negative for the presence of oocysts/sporocysts of *Sarcocystis*.

If we considered that the family Strigidae comprises 223 species of owls reported around the world, more studies are needed to elucidate the parasite fauna and involvement of these birds in the life cycles of parasites. Thus, new findings will help in increasing the knowledge about this interesting group of predators and their role as predators of rodents, which also act as intermediate hosts of several *Sarcocystis*.

## Conclusion

This work provided the first and the most comprehensive record on *Sarcocystis* from owls in Finland, thus highlighting the importance of molecular data in species identification. It also contributes to a better understanding of species diversity and the current taxonomic status of the unknown species within the genus *Sarcocystis*. This is the first time that the ITS1 region was sequenced for a *Sarcocystis* from owls as definitive hosts and clearly revealed the differences among species. Further works including examinations of owl populations, and particularly their prey in Finland, the Czech Republic, and worldwide, are required to elucidate the life cycle of the parasite.

## Data Availability Statement

The datasets presented in this study can be found in online repositories. The names of the repository/repositories and accession number(s) can be found below: https://www.ncbi.nlm.nih.gov/genbank/, MW349706, MW349707, MW373964, MW489293.

## Ethics Statement

The animal study was reviewed and approved by Centre for Economic Development, Transport and the Environment of Southwest Finland permit number VARELY/5933/2019.

## Author Contributions

OM conceived and designed the study. MK and EK conducted field research/collection. OM performed laboratory analyses and analyzed data. OM and DG-S wrote the main manuscript. All authors read and approved the final manuscript.

## Funding

Open access funding was provided by the Faculty of Agrobiology, Food and Natural Resources, Czech University of Life Sciences, Prague. The research project of owls in the Kauhava region was financially supported by the grant provided by the Regional Fund of the South Ostrobothnia of the Finnish Cultural Foundation.

## Conflict of Interest

The authors declare that the research was conducted in the absence of any commercial or financial relationships that could be construed as a potential conflict of interest.

## Publisher's Note

All claims expressed in this article are solely those of the authors and do not necessarily represent those of their affiliated organizations, or those of the publisher, the editors and the reviewers. Any product that may be evaluated in this article, or claim that may be made by its manufacturer, is not guaranteed or endorsed by the publisher.
